# A Sensor Fused Rear Cross Traffic Detection System Using Transfer Learning

**DOI:** 10.3390/s21186055

**Published:** 2021-09-09

**Authors:** Jungme Park, Wenchang Yu

**Affiliations:** College of Engineering, Kettering University, Flint, MI 48504-6214, USA; yu4416@kettering.edu

**Keywords:** ADAS, object detection, Convolution Neural Network, sensor fusion, rear cross traffic, radar, camera

## Abstract

Recent emerging automotive sensors and innovative technologies in Advanced Driver Assistance Systems (ADAS) increase the safety of driving a vehicle on the road. ADAS enhance road safety by providing early warning signals for drivers and controlling a vehicle accordingly to mitigate a collision. A Rear Cross Traffic (RCT) detection system is an important application of ADAS. Rear-end crashes are a frequently occurring type of collision, and approximately 29.7% of all crashes are rear-ended collisions. The RCT detection system detects obstacles at the rear while the car is backing up. In this paper, a robust sensor fused RCT detection system is proposed. By combining the information from two radars and a wide-angle camera, the locations of the target objects are identified using the proposed sensor fused algorithm. Then, the transferred Convolution Neural Network (CNN) model is used to classify the object type. The experiments show that the proposed sensor fused RCT detection system reduced the processing time 15.34 times faster than the camera-only system. The proposed system has achieved 96.42% accuracy. The experimental results demonstrate that the proposed sensor fused system has robust object detection accuracy and fast processing time, which is vital for deploying the ADAS system.

## 1. Introduction

Most traffic accidents occurred due to human error. Rear-end crashes are a frequently occurring type of collision, and approximately 29.7% of all crashes are rear-ended collisions [1]. Recent emerging automotive sensors and innovative technologies in computer vision enhance car and road safety. Advanced Driver Assistance Systems (ADAS) are intelligent systems that help drivers to avoid collisions and increase driving safety, such as Automated Emergency Braking (AEB), Blind Spot Detection (BSD), Lane Departure Warning (LDW), etc. ADAS are proven to reduce road fatalities by detecting obstacles in advance, generating warning signals for drivers, and controlling a vehicle accordingly.

A Rear-Cross Traffic (RCT) detection system is one of the ADAS applications, activated when a driver drives a vehicle backward. The RCT detection system warns the driver when obstacles are detected near the backing path. It is a challenging task because obstacles are approaching fast from the sides, which requires the system to react appropriately in a short time. The RCT detection system detects objects in blind spots or locations where obstacles are hard to be viewed through mirrors.

Currently, many commercial RCT detection systems are implemented using radar sensors. However, in many ADAS applications, using a single sensor is not enough for system accuracy. A radar sensor can detect object speed and range accurately and works under adverse weather conditions. However, the radar sensor often has too much noise and low resolution. Furthermore, a radar sensor is not able to classify the object types. On the other hand, the camera sensor has the advantages of low cost and high resolution. However, the camera sensor is susceptible to illumination changes. Therefore, the performance of an object detection system based on a camera sensor degrades easily under poor illumination conditions caused by sun glares or low illumination on rainy or foggy days [2]. Thus, an integration of an automotive radar sensor with a camera sensor is considered an efficient approach for an on-road obstacle detection system. Furthermore, since radar and camera sensors complement each other, combined information from two sensors can improve ADAS applications’ performance. In addition, horizontal field-of-view (FOV) is very important in the RCT detection scenario, and the system can achieve the wider FOV by combining more than two sensors. Normally, the camera sensor’s horizontal FOV is generally narrower than a radar sensor’s detection range. Therefore, by fusing the information from camera and radar sensors, the RCT detection system’s detection range is expanded, and the system accuracy can be improved.

**Figure 1:**
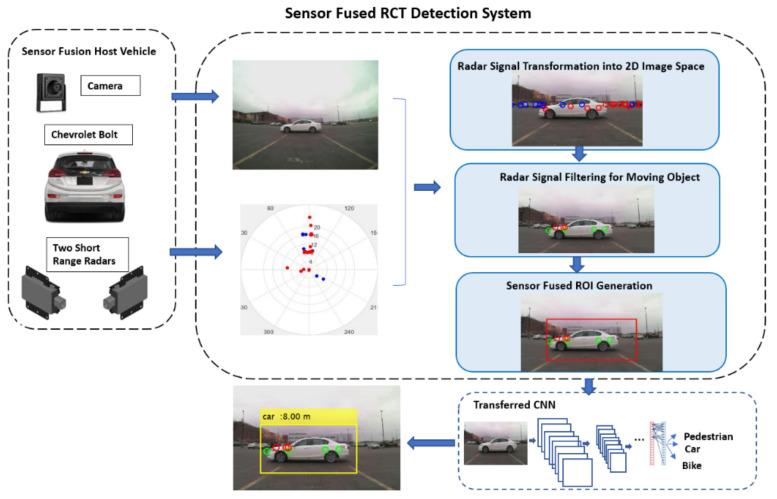
The overall architecture of the proposed RCT detection system.

Nowadays, many vehicles are equipped with a rearview camera, and it has also become a trend to mount short-range radars on the rear bumper for object detection in blind spots. This paper proposes a robust and cost-effective RCT detection system by fusing information from the rearview camera and short-range radars. The overall architecture of the proposed RCT detection system is presented in Figure 1. First, the proposed system combines signals from two radars mounted on the left and right sides on the rear bumper. Then, these combined radar signals are fused with the information from the camera sensor to detect the rear-end obstacles. Radar signals are transformed into an image coordinate system to fuse the information from the different sensors. Then, the proposed Region of Interest (ROI) extraction algorithm identifies target ROIs. Several Convolutional Neural Network (CNN) models are implemented using transfer learning technology to classify the object type. The identified ROIs in the camera image are fed into the transferred CNN model to classify the object type. Simultaneously, radar sensors provide the corresponding distance and speed of the detected object, which is critical information for collision avoidance.

In this paper, Section 2 presents a detailed literature review in the field of sensor fused object detection and the RCT detection system. Next, Section 3 presents a detailed methodology of the sensor fused RCT detection system. Then, Section 4 discusses the experimental results. Finally, we conclude the paper in Section 5.

## 2. Related Work

In camera-based image perception tasks, deep neural learning methods, especially CNNs, are popular and powerful techniques. For object detection in the automotive field, a Single Shot Detector (SSD) [3] has gathered many researchers’ interests due to its detection accuracy and speed. Meng et al. [4] made modifications to the original SSD model to improve detection performance, such as an image pyramid architecture for big objects detection and a strategy of image block method for small objects detection. In Zhang et al. [5], two additional deconvolutions and pooling layers were added to the SSD model in the feature pyramid. The proposed model in Zhang et al. [5], DP-SSD, has enhanced feature extractors that generate small bounding boxes for small objects with comparable accuracy and speed. Although these studies highlight the power of the camera and CNNs in object detection, depth information, one of the most important considerations in an automotive application, is not involved. In Guo et al. [6], through analyzing texture cues and blur cues from an image, depth information can be obtained. However, the depth information acquired by using only a camera sensor is not very accurate.

A radar sensor provides amplitudes, range, and velocity information for radar-related environment perception techniques to find obstacles. Lee et al. [7] proposed a purely radar-based system for object classification and recognition. A CNN model is utilized to distinguish objects based on their micro-Doppler signatures generated by the Doppler radar. However, they did not provide the details about the data they used in their experiments. Lombacher et al. [8] accumulate the radar data over several timestamps to develop radar grid maps for static object classification. Visentin et al. [9] present a post-processed range-velocity map fed to the CNN model for object classification, and Kim et al. [10] complete moving object classification by a series of radar range-velocity maps and CNN. However, those classification methods based on only radar sensors did not provide promising results. In addition, most of the experiments were conducted either in-door or in simulation environments.

Bi et al. [11] presented a method of coordinate conversion between radar and camera coordinate systems for radar-camera sensor fusion techniques. The radar signals are projected onto camera images using the proposed method. However, they assumed that the object type is known for the ROI generation using different widths and heights for vehicles and pedestrians. Hyun et al. [12] use a vehicle rooftop camera and a radar mounted on the front bumper to find the target object. However, the ROI information (range, radial velocity, and angle) is not the bounding box information in the proposed system. The authors did not provide details on finding the object’s bounding box information as ROI. Chadwick et al. [13] proposed a method that uses one Doppler radar and two cameras. Two cameras have different focal lengths. The one with a short focal length is used to observe wider FOV and the other with a long focal length is responsible for acquiring distant object information. The fused radar scans with camera images are used for object detection by a neural network to improve performance. Among those radar-camera sensor fusion publications, very few strategies are solving vehicle rear-end scenarios. A safe lane changing method is proposed in Kim et al. [14], based on radar and vision sensor fusion. They applied a CNN model to the rearview to detect objects. Then, radar signals are fused with the detections. For target tracking and motion path prediction, a Kalman filter is applied.

Among these publications, a significant portion is related to the radar-camera sensor fusion algorithm to detect the objects in front. However, very few strategies are solving vehicle rear-end-related problems. Huang et al. [15] proposed a stereo vision-based obstacle detection system focused on reverse gear driving scenarios. In their proposed method, the obstacles can be detected based on a disparity map with depth information. However, because the proposed method depends on the depth disparity map, errors can easily occur in calculations when the disparity map is not obvious. As Takahashi et al. [16] mentioned in their survey, people often feel nervous when backing out due to the blind spot at the driver’s side. Since backing out is the most common parking style in North America because of the wide parking space and the purpose of loading cargo easily, the RCT detection system assists drivers when backing from a 90-degree parking spot or a 60-degree parking spot. In a cross-traffic scenario, the object’s motion path is defined as crossing from one side of the host vehicle to the other side of the host vehicle (perpendicular to the host vehicle’s moving path). Further, the moving objects usually approach from the driver’s blind spot. By considering these requirements in the RCT detection system, expanded FOV for object detection is desirable.

## 3. The Rear Cross Traffic Detection Methodology

Radar and camera sensors complement each other. A combination of these two sensors is ideal for building a robust RCT detection system. The radar sensor provides useful data about the obstacles, such as distance, angle, and velocity signals. That information is important for the host vehicle to mitigate and avoid any possible collision. However, radar signals cannot accurately classify the object type because signals are low-resolution and noisy. On the other hand, the camera sensor classifies object types by using the deep CNN model. However, the deep CNN-based object detection model requires a high computational cost to find object locations in an image. The high computational cost makes it difficult to be deployed on real-time applications. Besides, the system’s performance based solely on the camera sensor degraded severely with adversary weather conditions because the camera sensor is sensitive to illumination changes. Because of these reasons, sensor fusion is clearly the most effective way to improve any ADAS as the data from different sensors complement each other, making the system robust. This paper proposes a robust sensor fused RCT detection system by combining the information from two radars and a wide-angle camera sensor.

### 3.1. Hardware Set-Up for the RCT Detection System

**Figure 2:**
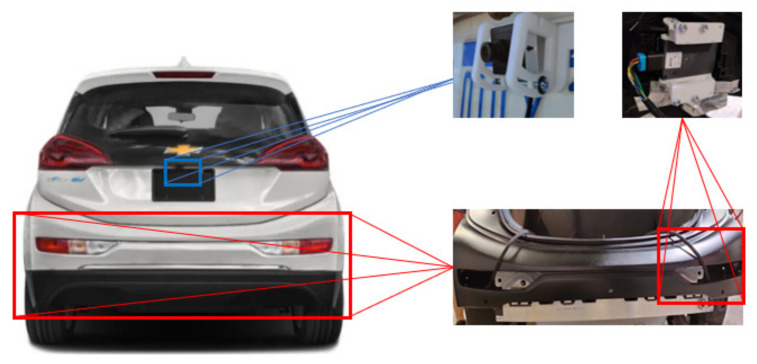
Hardware setup for the RCT detection system.

To build the proposed sensor fused RCT detection system, two Delphi SRR radars (Aptiv, Troy, MI, USA) and one Spinel camera with a 2.1 mm Sony IMX179 lens (Spinel, Newport Beach, CA, USA) are selected [17]. The radars are mounted on the rear bumper’s left and right sides to detect objects behind and on the host vehicle’s sides. The camera is mounted on the rear license plate, under the lid of the rear windshield, and above the rear bumper, as shown in Figure 2.

**Figure 3:**
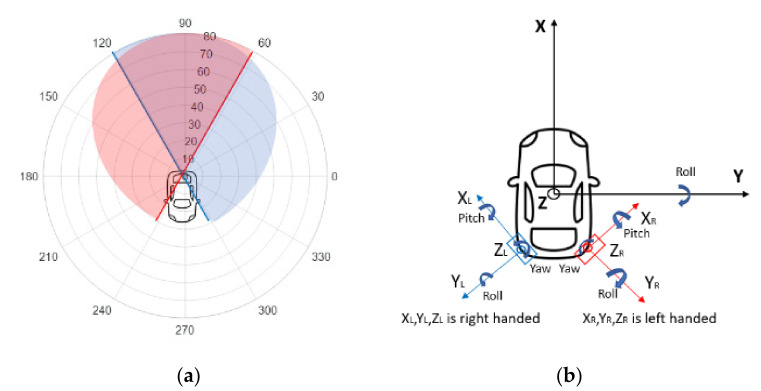
(**a**) Radar system FOV: 10 m per range grid, 30° per angle grid; (**b**) radar alignment illustration.

The radar system consists of two rearward-looking single beam mono-pulse radars located at each corner of the vehicle to detect objects behind and to the side of the host vehicle. Each radar can cover 180° FOV horizontally. The left and right radars have some overlapped FOV and the combined radar system has 300° FOV, as shown in Figure 3a. One radar is a right-handed radar and the other one is a left-handed radar. Further, the two radars need to be installed with an angle of 30 degrees (+30 yaw of the vehicle travel direction), as presented in Figure 3b.

**Figure 4:**
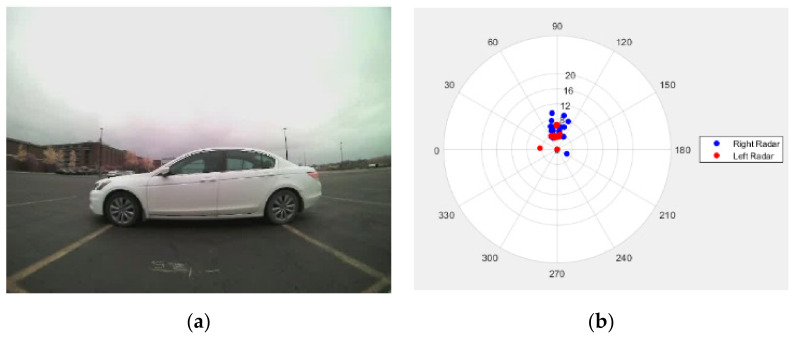
Validation of radar signals: (**a**) The test vehicle is placed on the pre-measured location at the center of the rear bumper; (**b**) red dots are signals from the left radar and blue dots are signals from the right radar.

The signals from two radars are collected using CAN bus messages simultaneously. The signals utilized are amplitude, angle, range, range rate, and validity level. The definitions of these signals are as follows. The amplitude signal represents the millimeter-wave reflectivity of detected object surfaces. The angle signal is the measured angle from the detected object to the centerline of the radar. The range information represents the distance from the detected object to the radar. The range rate shows the changing rate of range information, which is useful for tracking a moving object. Finally, the validity level represents the validity level of the collected data. If the validity level value is high, the radar is confident about the detected object. To validate the radar signals from two radar sensors mounted on the rear bumper, a testing vehicle is placed on the pre-measured location at the center of the rear bumper, as presented in Figure 4a. The signals captured by two radar sensors are plotted in Figure 4b. The blue dots are signals from the right radar, and the red dots are signals from the left radar. Both left and right radars captured the white vehicle near the 90° axis.

### 3.2. The Sensor Fused RCT Detection System

The proposed RCT detection system consists of several main modules, as presented in Figure 1. First, the radar signals are transformed into a 2D camera image coordinate system. The projected radar signals onto 2D image space are utilized to determine the possible object locations in the image. The transformed radar signals are filtered out based on speed, range, and validity level information to detect moving objects. Since the radar signals are several points on the target object (low-resolution) and often contain noisy signals, it is difficult to have the whole contour of an object solely rely on the radar signals. The proposed sensor fused ROI extraction algorithm finds the candidate ROIs in the given image accurately. Finally, the identified candidate ROI is fed into a CNN classifier to determine the object type.

#### 3.2.1. Coordinate Transformation and Radar Signal Filtering

To fuse the information from the camera and the radar sensors, the radar signals’ polar coordinates are transformed into the world coordinates and projected onto the camera images. To calculate the transformed coordinate (*u*, *v*) from the given radar signals, the transformation matrices adapted from [11] are presented in Equations (1) and (2).

(1) $$\left[\begin{array}{c} X_{c}\\ Y_{c}\\ Z_{c}\\ 1 \end{array}\right]=\left[\begin{array}{cccc} 0 & -1 & 0 & L_{x}\\ 0 & 0 & 0 & L_{y}\\ 1 & 0 & 0 & L_{z}\\ 0 & 0 & 0 & 0 \end{array}\right]\left[\begin{array}{c} r\cos\theta \\ r\sin\theta \\ 0\\ 1 \end{array}\right]$$

where $L_{x},\;L_{y},$ and $L_{z}$ are the distances between radar and camera in the *x-*, *y-*, and *z-axis* direction, respectively. According to the hardware set-up in this project, $L_{x}\;$= 0.7507 m, $L_{y}\;$= 0.2413 m, and $L_{z}$= 0 m. In Equation (1), the corresponding camera coordinate system ($X_{c}$_,_ $Y_{c}$_,_ $Z_{c}$) is calculated using the given radar range, *r*, and angle signal, *θ*. Using Equation (2), those signals are transformed into camera image coordinates (*u*, *v*) and projected onto an image.

(2) $$Z_{c}\left[\begin{array}{c} u\\ v\\ 1 \end{array}\right]=\left[\begin{array}{ccc} \frac{1}{d_{x}} & 0 & u_{0}\\ 0 & \frac{1}{d_{y}} & v_{0}\\ 0 & 0 & 1 \end{array}\right]\left[\begin{array}{cccc} f & 0 & 0 & 0\\ 0 & f & 0 & 0\\ 0 & 0 & 1 & 0 \end{array}\right]\left[\begin{array}{c} X_{c}\\ Y_{c}\\ Z_{c}\\ 1 \end{array}\right]$$

where symbols used in Equation (2) are defined in Table 1.

In the proposed RCT detection system, a wide horizontal FOV 180° is used, so the $d_{x}\;\mathrm{and}\;d_{y}$ values are adjusted empirically. Adjustment for $d_{x}$ is made using Equation (3) for the given radar range, *r*, and angle signal, *θ*. First, a radar reflector is placed in the center of the camera image and the radar signals on the reflector are recorded. The location of the radar reflector in the x-direction is *u* = 345 and the corresponding radar range, *r*, and angle, *θ*, signals for the reflector are *r* = 6.5 m and *θ* = 60°. Using Equation (3), the value of $d_{x}$ is calculated as $1.07\times 10^{-5}$. Similarly, the value of $d_{y}$ is also calculated.

(3) $$d_{x}=\frac{\left(L_{x}-r\times \sin\theta \right)\times f}{r\times cos\theta \times \left(u-u_{0}\right)}.$$

**Figure 5:**
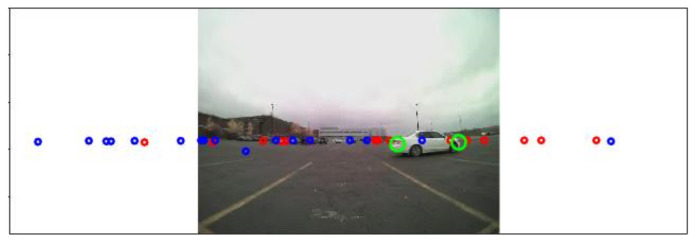
Projected radar points over the corresponding image frame and beyond of the camera image.

Since the FOV covered by two radar sensors is much wider than the FOV of the camera sensor, 300° versus 180°, some radar signals are located outside of the images. Figure 5 presents the expanded areas, such as 400 pixels on the left and 400 pixels on the right side. Those expanded areas hold additional information about the approaching obstacles even though they do not contain any corresponding camera pixel information. The expanded areas can be utilized as early approaching warning signals before obstacles enter the camera FOV.

**Figure 6:**
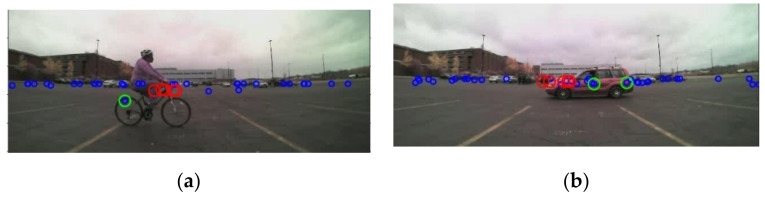
Static and moving points separated and projected: (**a**,**b**) The small blue dots represent static objects. The green and red circles represent the radar signals for moving objects from left and right radar, respectively.

After the signals from two radars are successfully transformed into the image coordinate system, then moving objects can be identified using the range rate information from the radar sensor. The range rate information is useful to identify a moving object. In the proposed RCT detection system, the primary goal is to detect all moving objects with speed > 0.1 m/s (equals to 0.36 km/h), and the range is within a 30 m range. Therefore, valid radar signals have a range value less than 30 m, range rate > 0.1 m/s (equals to 0.36 km/h), and validity level ≥ 1. In Figure 6, the small blue dots represent static objects. The green circles represent the radar signals for moving objects obtained from the left radar. The red circles are the radar signals for moving objects obtained from the right radar.

#### 3.2.2. The Proposed ROI Extraction Algorithm

Because the radar signals cannot classify the object type due to the low-resolution information, the ROI extraction is a challenging task. However, if the ROIs for object detection are identified correctly, this reduces the processing time tremendously by removing the step for scanning a whole image using a sliding window with different sizes for searching the potential object in the image. In this paper, a sensor fused ROI extraction algorithm is developed. The procedures for the proposed ROI extraction algorithm are explained below:

Step 1: Find a list of radar signals that belongs to the same object

The radar information, $R_{i},$ contains the converted coordinates in 2D image space, (*x*, *y*), range, *r*, range rate, v, and angle, θ:

(4) $$R_{i}=\left\{\left(x,y\right),r,\;v,\;\theta \right\},\;\mathrm{where}\;i=1,\;2,\text{…},\;k.$$

Each signal in $R_{i}$ is represented using the ‘.’ notation such that the notation $R_{i}.r$ represents the range signal in $R_{i}$. For each radar information, $R_{i}$, the bounding box, $BB_{i}$ = {($x_{1},\;y_{1}),\;\left(x_{2},\;y_{2}\right)$} is defined as reverse proportional to the range, $R_{i}.r$:

(5) $$\alpha =80-R_{i}.r$$

(6) $$BB_{i}.x_{1}=R_{i}.x-\alpha ,\;BB_{i}.y_{1}=R_{i}.y-\alpha$$

(7) $$BB_{i}.x_{2}=R_{i}.x+\alpha ,\;BB_{i}.y_{2}=R_{i}.y+\alpha$$

where the coordinates ($x_{1},\;y_{1})$ and ($x_{2},\;y_{2})$ are the top left point and the bottom right point in the bounding box,$\;BB_{i}$, respectively. On the other hand, the parameter α is reverse proportional to the range in $R_{i}.$ Step 2: Merging bounding boxes

Two bounding boxes, $BB_{i}$ and $BB_{j}$ will be merged if the Intersection Over Union (*IOU*) is greater than 0.5. The $IOU\left(BB_{i},\;BB_{j}\right)$ is a ratio of the area of intersection of two bounding boxes to the area of the union of them.

(8) $$IOU\left(BB_{i},\;BB_{j}\right)=\frac{BB_{i}\;\cap \;BB_{j}}{BB_{i}\cup \;BB_{j}}.$$

**Figure 7:**
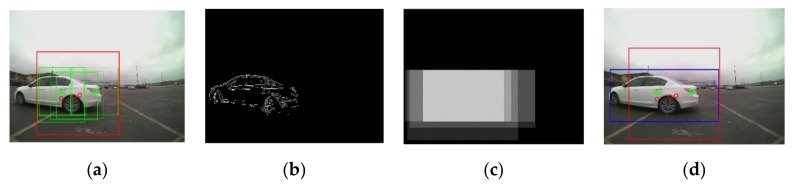
The proposed ROI generation algorithm: (**a**) the green bounding boxes are generated from the radar signals, the merged ROI is presented with the red square; (**b**) the first gradient of the motion image after binarization; (**c**) the temporal correlation map; (**d**) redefined ROI in the blue rectangular using the temporal correlation map.

**Figure 8:**
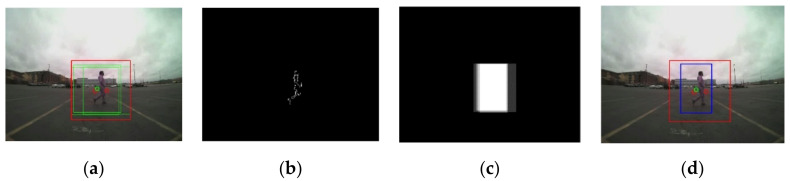
Refined ROI result from the proposed ROI generation algorithm: (**a**) the green bounding boxes are generated from the radar signals, the merged ROI is presented with the red square; (**b**) the first gradient of the motion image after binarization; (**c**) the temporal correlation map; (**d**) redefined ROI in the blue rectangular using the temporal correlation map.

At the end of Step 2, all bounding boxes that belong to the same object are merged into one, becoming one ROI for the object, as shown in Figure 7a and Figure 8a. In Figure 7a and Figure 8a, the green square box represents the bounding box, $BB_{i,\;}$ generated by the radar signal, $R_{i},\;i=1,\ldots ,k$. After the merging process, the merged ROI is presented in the red square. Due to the low resolution and noise in the radar sensor information, the defined ROIs in Step 2 are not accurate enough in many cases. Therefore, the roughly estimated ROIs in Step 2 have been further adjusted by utilizing the image information from the camera sensor in Step 3.

Step 3: Update the ROIs using temporal correlation in the video frames

For each $ROI_{k}$ = {($x_{1},\;y_{1}),\;\left(x_{2},\;y_{2}\right)\;$}, *k* = 1,..., *p*, the motion matrix, $M_{t}$, is calculated using the intensity differences between the previous frame, $Img_{t-1}$, and the current image frame, $Img_{t}$.

(9) $$\forall \left(x,y\right)\;\mathrm{where}\;x\in [x_{1}-\beta ,\;x_{2}+\beta ],\;y\in [y_{1}-\beta ,\;y_{2}+\beta ],\;M_{t}\left(x,y\right)=\left|Img_{t}\left(x,y\right)-Img_{t-1}\left(x,y\right)\right|.$$

In the motion matrix, $M_{t}$, the offset, β = 20, is added to the ROI defined in Step 2. The offset is added to cover the ROI shift between the previous frame, $Img_{t-1}$, and the current frame $Img_{t}$. With the expanded ROI, the motion is calculated using Equation (9). The motion output that exceeds the range of [0, 255] is truncated and the first gradient of $M_{t}$ is calculated. By thresholding the first gradient of $M_{t}$, a binary image is generated, as shown in Figure 7b and Figure 8b. Since the frame rate in the camera sensor is 30 frames per second, contents in consecutive frames are highly correlated. The object locations are overlapped within small consecutive frames (e.g., 5 frames) due to the temporal correlation in consecutive image frames [18]. The final $ROI_{k}$ is determined by tracing the locations of $ROI_{k}$ in the previous frames (e.g., 5 frames). The accurate final $ROI_{k}$ is found using the temporal correlation map. The temporal correlation map also solves the problem of missed radar signals between image frames.

The plots in Figure 7 and Figure 8 present the steps of the proposed sensor fused ROI generation algorithm.

#### 3.2.3. Object Classification Using the Transferred CNN Model

**Figure 9:**
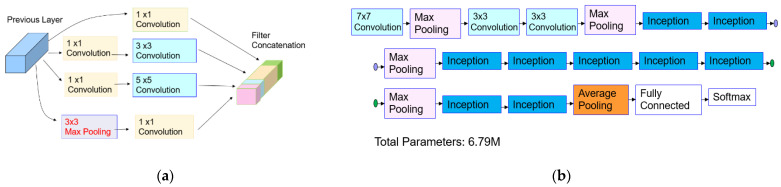
GoogLeNet: (**a**) inception module in GoogLeNet [23]; (**b**) summary of GoogLeNet architecture.

Object classification refers to a task that identities the category of the object belongs to. In many ADAS applications, different control signals are generated depending on the identified object type, such as car, pedestrian, bike, etc. Since AlexNet [19] achieved an outstanding improvement on the ImageNet Large Scale Visual Recognition Challenge (ILSVRC) in 2012, the state-of-the-art CNN models have achieved a remarkable breakthrough in object classification. Those CNN models were trained with 1.2 M image data samples to classify 1000 classes. Those CNN models are AlexNet [19], VGG-16 [20], VGG-19 [20], DarkNet [21], Resnet-50 [22], and GoogLeNet [23], etc. The architectures of those CNN models are summarized in Table 2 and Figure 9. The GoogLeNet architecture contains the “Inception” module presented in Figure 9a. The inception module improves the computational cost by adding a 1 × 1 convolutional layer, which reduces the output dimension. The dimension reduction allows for a gain of computational efficiency and ensures the capability of a deeper and wider network. The overall architecture of GoogLeNet is displayed in Figure 9b.

**Figure 10:**
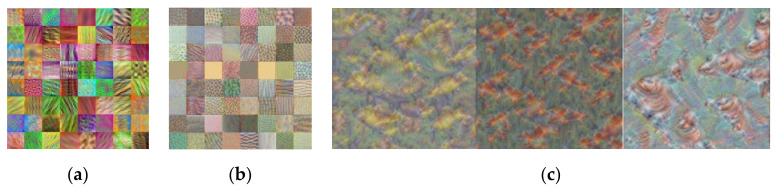
Features learned in GoogLeNet: (**a**) the first 64 features extracted in the 2nd convolution layer; (**b**) the first 64 feature output in the 1st inception module; (**c**) the first three outputs in the last fully connected layer.

It is a challenging task to develop an object classification system with a relatively small dataset. In general, the neural network trained with a small number of data samples is prone to poor performance and overfitting. However, by utilizing the state-of-the-art CNN models in Table 2 and Figure 9, which were trained with a large amount of data, those learned features could be transferred to a new system with a smaller dataset. Transfer learning is a machine learning method that reuses those pre-trained CNN models as a starting point. For example, those CNN models learned the image’s shapes, edges, and lighting in the lower layers with visual image data presented in Figure 10a,b. Because these features are generalized across most types of images, utilizing those trained features to the new task with the relatively small data provides an overall better accuracy than training a new model from scratch. In Figure 10c, the first three outputs in the last fully connected layer are presented. Those three outputs are strongly activated to the corresponding class.

**Figure 11:**
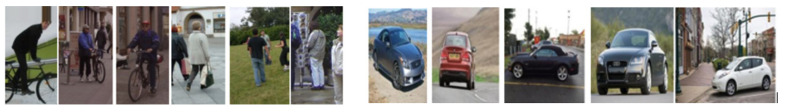
Training data sample images from [24,25,26,27].

**Figure 12:**
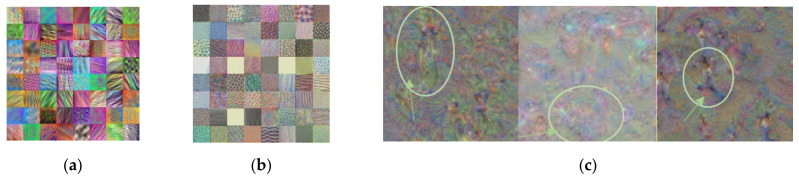
Features learned in GoogLeNet: (**a**) the first 64 features extracted in the 2nd convolution layer; (**b**) the first 64 feature output in the 1st inception module; (**c**) the three outputs in the last fully connected layer.

To develop the object classification system inside the proposed RCT detection system, the training data were collected from [24,25,26,27]. In total, 8044 training image samples were collected, including 2651 samples for the bike class, 3381 samples for the car class, and 2012 for the pedestrian class. The sample images of the training dataset are given in Figure 11. Using the collected data, six state-of-the-art CNN models in Table 2 and Figure 9 are retrained using transfer learning. Figure 12 demonstrates the extracted features after transfer learning of GoogLeNet. In Figure 12a,b, the features transferred in the low level of the network are very close to the original features in Figure 10a,b. This is because most of the original features are reused in the transferred system in the lower layers of the network. The channel output images in Figure 12c represent the selected classes such that the channel image for the ‘bike’ class contains distinct wheels of the bike, the channel image for the ‘car’ class contains the shape of vehicles, and the pedestrian shape is represented in the channel output for the pedestrian class. Thus, it demonstrates that transfer learning is completed successfully.

The training dataset is divided into 90% for training and 10% for validation during the training. The validation results in Table 3 show that VGG19, GoogLeNet, and VGG 16 have the classification accuracy of 97.01%, 96.89%, and 96.52%, respectively, with the learning rate α = 0.0001. In Table 3, the performances with the smaller learning rate α = 0.0001 are better than the performances with α = 0.0002 in general. Transfer learning comes with a variety of benefits, other than just helping improve the performance of a small dataset. It also saves time during training. Because fewer data are required and low-level features are already learned in the pre-trained CNN models, only a few weights need to be updated during the training process for the new system. In Table 3, the longest training time is about 6.5 h taken to train VGG19.

## 4. Experiments on the RCT Detection System

**Figure 13:**
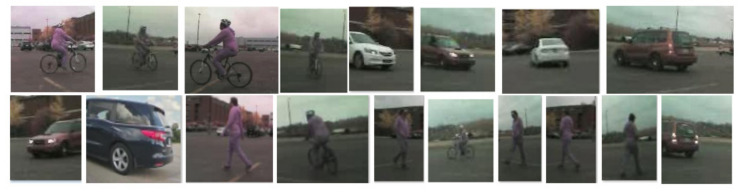
Sample image patches used to test the transferred CNN models.

The transferred CNN models are tested for the classification performance on the dataset collected for the proposed RCT detection system. The testing data samples contain a total of 12,807 samples, including 4796 samples for the ‘bike’ class, 3332 samples for the ‘car’ class, and 4679 for the ‘pedestrian’ class. Figure 13 shows the sample image patches used to test the transferred CNN models. The classification results generated by the transferred CNN models are summarized in Table 4. Overall, three transferred CNNs, VGG-19, GoogLeNet, and VGG-16 generate good performances on the test dataset. The average accuracies of the top three models, VGG-19, GoogLeNet, and VGG-16, are 96.42%, 96.17%, and 95.04%, respectively.

**Figure 14:**
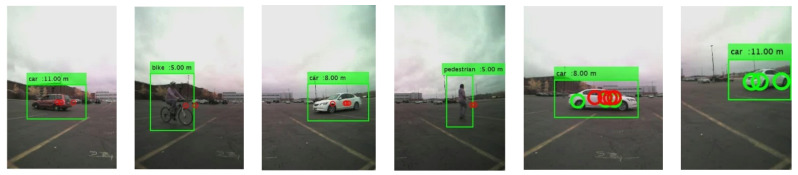
Example images of object detection results.

**Figure 15:**
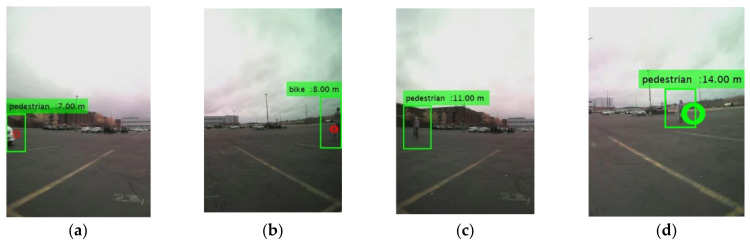
False classification examples: (**a**,**b**) false classification because the shape of the objects is partially presented; (**c**,**d**) false classification because the shape of the bike looks similar with the shape of pedestrian.

In the proposed RCT detection system, the proposed sensor fused ROI extraction algorithm finds the ROIs using the radar signals first. Then, the ROIs are adjusted correctly using the motion and the temporal correlation information in the consecutive image frames. Finally, the identify ROIs are resized to the input image size of the trained CNN model and fed into the CNN model. The CNN model classifies the object type, and the matching radar signals provide the distance to the object from the host vehicle, as presented in Figure 14. Several detection results are presented in Figure 14. From left to right, the target classes are car, bike, car, pedestrian, car, and car, respectively. In the RCT detection system, most of the classification errors have happened in two cases. In Figure 15a,b, when the object is located in the boundary of the images, the full shape of the object is not presented yet in the image. Thus, the classification result is not accurate. The other case presented in Figure 15c,d is when the object is at a larger distance from the host vehicle. Since the object is located far away from the camera sensor, its shape is becoming smaller, and it becomes similar to the shape of another object.

To compare the performance of the sensor fused RCT detection system with the camera-only detection system, a camera-only detection system is implemented using the Faster R-CNN architecture [28]. The Faster R-CNN architecture contains the convolutional layers as the feature extraction network. The top three CNN models in Table 4 are used as the feature extraction network inside the Faster R-CNN model. The vehicle detection systems with various architectures are trained with the Udacity vehicle dataset [29] that contains 8738 images with labeled data. The different architectures of the vehicle detection systems are evaluated with the vehicle dataset of 6233 image frames collected in the RCT detection system. The performance of each vehicle detection architecture is measured with two metrics, precision and recall. The two metrics are defined as follows: precision = TP/(TP + FP), recall = TP/(TP + FN), where TP = True Positive, FP = False Positive, and FN = False Negative.

The experimental results of the various rear cross vehicle detection systems are presented in Table 5. The best architecture for the sensor fused RCT detection system is VGG19, which has 0.9798 precision and 0.9668 recall. The average processing time of the VGG-19 based architecture is 0.0057 s, including the time for the ROI detection by the radar sensors. The average processing time is measured using the Dell G7 laptop computer with the Intel^®^ Core™ i7 processor, 16GB memory, and the NVIDIA GeForce GTX 1060. For the camera-only system, the best performance is generated by VGG-16faster R-CNN, which has 0.9986 precision and 0.8589 recall. The camera-only systems based on the faster R-CNN have low recall rates because the CNN-based object detection systems have poor performances in detecting small size objects. The average processing time of the VGG-16faster R-CNN is 0.0931 s.

The proposed sensor fused RCT system achieved better detection performance and reduced the processing time. The sensor fused system based on VGG19 is 19.53 times faster than the processing time of the VGG-19faster R-CNN and 16.34 times faster than the processing time of the VGG-16faster R-CNN. In the camera sensor-only system, the trained CNN model scans an image using anchor boxes to find the possible locations of objects and classify the object type if the location was determined to contain the object. This detection process requires a high computational cost and makes it difficult to be deployed. On the other hand, in the proposed sensor fused RCT detection system, the sensor fused ROI generation algorithm detects all possible targets by combining radar signals and information from the camera images. The identified ROIs are fed into the CNN model to classify the object type. The proposed algorithm reduces the object detection time tremendously by removing the step for scanning the whole image.

**Figure 16:**
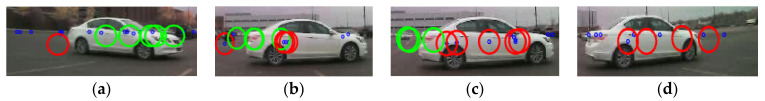
Tracking robustness by two radar sensors: (**a**) the left radar (more green circles than the red circles) mostly captures the object; (**b**,**c**) the signals from the left and right radar sensors detect the object; (**d**) only signals from the right radar are captured the object.

In addition, the FOV of the system is extended using the proposed system. Because two radars are implemented in the proposed system, radars’ horizontal FOV is greatly expanded. Comparing to one radar installed in the middle of the rear bumper, two radars installed on the sides of the rear bumper have a horizontal FOV of 300° (with some overlapped region in the middle), whereas a single radar only has a horizontal FOV of 180°. So, more information can be captured by an expanded FOV. It is observed that the object tracking by two radar sensors is robust, as presented in Figure 16. In Figure 16, the left radar points are represented by green circles, and the right radar points are indicated by red circles. As shown in Figure 16a, the object is on the left side of the host vehicle, and the left radar (more green circles than the red circles) mostly captures the object. In Figure 16b,c, the object is moving toward the middle of the image, and the signals from the left and right radar sensors detect the object. On the other hand, in Figure 16d, the object is on the right side of the host vehicle, and only signals from the right radar are observed on the image.

## 5. Conclusions and Future Scope

In this paper, a robust sensor fused RCT detection system has been proposed using two radars and one wide-angle camera. The novelties of the proposed RCT detection system are summarized as follows. (1) The integration of the radar and camera sensors for the RCT detection system. As far as sensor fusion is concerned, the radar points are mapped to camera images by transforming the radar coordinate system into the image coordinate system. Furthermore, the static and moving objects are distinguished using the information from the two radar sensors on the overlay image. (2) The new sensor fused ROI extraction algorithm is developed by fusing the information from the two radar signals and the corresponding image data. The proposed sensor fused ROI extraction algorithm accurately identified the ROIs of the objects. The proposed algorithm makes the processing time 15.34 times faster than the processing time of the camera-only system by avoiding scanning the whole image for the possible locations of the object. The identified ROIs are fed into the CNN model for object classification. (3) We carried out experiments on transfer learning for the object classification task. Regarding CNN object classification, six CNN models have been selected for comparison studies. Through experiments, the trained system can differentiate vehicles, bikes, and pedestrians in the image frame at different levels of accuracy. Three transferred CNN models, VGG-19, GoogLeNet, and VGG-16, have good performances on the RCT detection system dataset. The average accuracy of the top three models, VGG-19, GoogLeNet, and VGG-16, are 96.42%, 96.17%, and 95.04%, respectively. (4) The experiments for the comparison between the proposed sensor fused system and the camera only system. A comparison between the sensor fused RCT system with the camera-only system has been conducted. The proposed sensor fused RCT detection system reduced the processing time and was 15.34 times faster than the camera-only system. The experimental results demonstrate that the proposed sensor fused system has robust object detection accuracy and short processing time, which is vital for deploying the ADAS system.

For future research scopes, comparison tests can be carried out between two radars on the sides of the rear bumper and one radar in the middle of the rear bumper. In addition, further research on the embedded programming for real-world implementation is required.

## Figures and Tables

**Table 1:** Parameters to find the transformed coordinate (*u*, *v*).

Symbol	Description	Value
*d_x_*	Physical x pixel length in the image coordinate	-
*d_y_*	Physical y pixel length in the image coordinate	-
*u*_0_	x pixel coordinate of the intersection point between axis $Z_{c}$ and image plane	640
*v*_0_	y pixel coordinate of the intersection point between axis $Z_{c}$ and image plane	480
*f*	Camera focal length	0.0021 m
*u*	x pixel coordinate of radar detection plotted on the image	-
*v*	y pixel coordinate of radar detection plotted on the image	-

**Table 2:** Architectures of the state-of-the-art CNN models.

Layer	AlexNet	VGG-16	VGG-19	DarkNet	ResNet-50
Convolution	5	13	16	19	49
Max Pooling	3	5	5	5	1
Avg. Pooling	-	-	-	1	1
Fully Connected	2	3	3	-	1
Softmax	1	1	1	1	1
Parameters (Millions)	62 M	138 M	144 M	20.8 M	25.5 M

**Table 3:** Training results for object classification.

CNN Models	Training Time for Transfer Learning (min)		Validation Accuracy (%)	
	α = 0.0001	α = 0.0002	α = 0.0001	α = 0.0002
AlexNet	10	8	93.28	92.41
VGG-16	115	115	96.52	95.40
VGG-19	390	385	97.01	95.65
Darknet-19	50	49	87.81	87.56
Resnet-50	57	57	93.28	93.91
GoogLeNet	27	27	96.89	96.89

**Table 4:** Testing results for object classification by the transferred CNN models.

Class	Accuracy (%) per Class Type			Overall Accuracy (%)
	Bike	Car	Pedestrian	
AlexNet	92.91	87.48	99.17	93.78
VGG-16	92.47	97.72	95.77	95.04
VGG-19	96.96	94.60	97.16	96.42
Darknet-19	88.05	78.72	90.55	86.54
Resnet-50	95.23	93.91	78.31	88.70
GoogLeNet	94.83	95.77	97.82	96.17

**Table 5:** The performance comparison: sensor fused RCT detection system vs. camera-only detection system.

Vehicle Detection System		Processing Time per Fame (s)	Precision∗100	Recall∗100
Sensor Fused detection System	VGG-16	0.0052	97.97	96.32
	VGG-19	0.0057	97.98	96.68
	GoogLeNet	0.0047	97.97	96.20
Camera only detection system:	VGG-16faster R-CNN	0.0931	99.86	85.89
	VGG-19faster R-CNN	0.1170	98.81	81.84
	GoogLeNetfaster R-CNN	0.4095	98.64	80.37
